# Familial Case of Cherubism from South India: Differential Diagnosis and Report of 2 Cases

**DOI:** 10.1155/2014/869783

**Published:** 2014-12-08

**Authors:** Varun Muthuraman, Soundarya Srinivasan

**Affiliations:** ^1^Department of Oral and Maxillofacial Surgery, Best Dental Science College & Hospital, No. 69/1-A, Melur Road, Kodikulam, Madurai, Tamil Nadu 625104, India; ^2^Department of Oral and Maxillofacial Pathology, Best Dental Science College & Hospital, No. 69/1-A, Melur Road, Kodikulam, Madurai, Tamil Nadu 625104, India

## Abstract

Cherubism is a rare familial multilocular cystic lesion of the jaws. The condition clinically appears as a bilateral symmetric swelling of the cheeks in children and is the primary reason for referral. It is a rare lesion of the jaws that has a dominant pattern of inheritance. We report two cases of cherubism, that of a boy and his mother suggestive of a strong familial incidence. A variety of lesions of the jaw mimic this condition and hence the differential diagnosis has been emphasised.

## 1. Introduction

Cherubism was first reported by Jones et al. in 1952 [1]. The term cherubism is derived from the word “cherub” which means angels with childish full cheeked face gazing upwards as if eyes look up to “heaven.” A mutation in the gene SH3BP2 has been demonstrated [2]. The lesion is almost always of a benign nature and only produces a cosmetic deformity in childhood and starts regressing after puberty. However, a rare fatal case [3] of cherubism has been reported in literature. The treatment is mainly conservative. Invasive surgical procedures are avoided and a wait and watch approach is the mainstay in the treatment protocol.

## 2. Case Report

**Figure 1:**
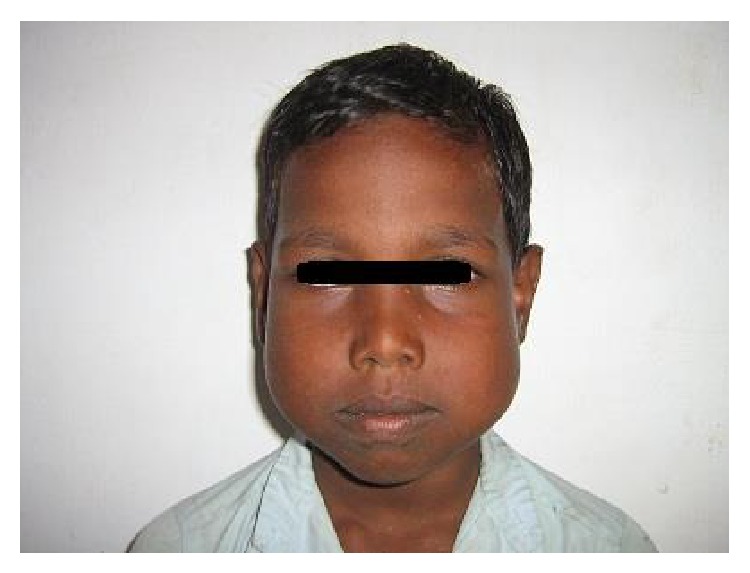
Facial view showing bilateral swelling of mandible.

**Figure 2:**
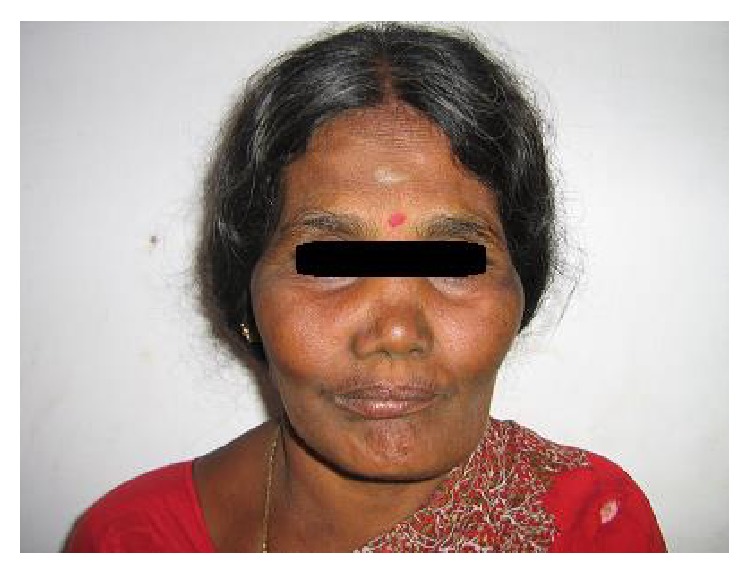
Facial view showing bilateral swelling of maxilla and zygoma.

**Figure 3:**
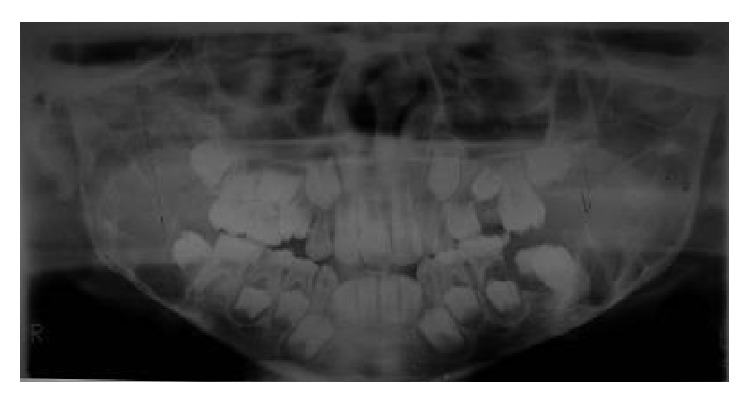
Orthopantomogram showing bilateral multilocular radiolucencies involving posterior body, ramus of mandible, and condyle typically spared.

**Figure 4:**
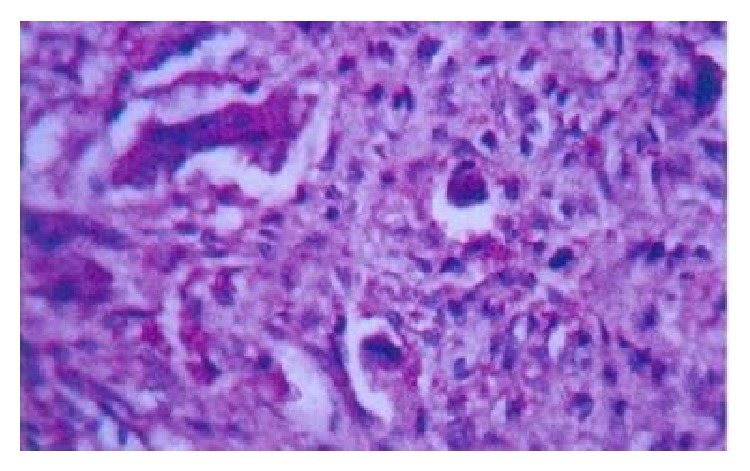
Histopathological section showing multinucleated giant cells resembling osteoclasts, scattered in a fibrous connective tissue stroma.

A young boy aged 9 yrs reported with his mother with a presenting complaint of bilateral cheek swelling. Familial history suggested that his mother had undergone surgical correction for a similar swelling of the jaws when young. The boy was the younger of the two siblings. His elder brother did not have any peculiar features. Clinical examination of the boy showed symmetrical and bilateral swelling of his cheek and mandible (Figure 1). He was classified by Seward and Hankey [4] (1975) as first degree (bilateral lesions confined to the lower molar regions and posteriorly up to the coronoid process). On palpation, the bilateral swelling was a bony hard swelling and nontender. Clinical examination of the boy's mother also showed bilateral enlargement of the maxilla, zygoma, and mandible (Figure 2). Investigation of the boy with an OPG (Figure 3) showed bilateral multilocular radiolucent lesions of the posterior body of mandible and ramus. However, the condyle was spared. Multiple carious teeth were present on intraoral examination. OPG also demonstrated abnormalities in the eruption status of permanent teeth and displaced mandibular second molar tooth buds. Blood chemistry was performed to rule out primary hyperparathyroidism. The histopathologic examination of the lesion revealed numerous multinucleated giant cells resembling osteoclasts, scattered in a fibrous connective tissue stroma (Figure 4). Final diagnoses of cherubism were made. His mother was reassured about the benign course of the condition and regular follow-up of the boy was advised.

## 3. Discussion

Cherubism is a rare giant cell lesion of bone affecting children. Several family reports from different countries have clarified cherubism as a hereditary bone disease [5].

The differential diagnosis of cherubism includes fibrous dysplasia of the jaws [6], central giant cell granuloma [7], brown tumour, true giant cell tumour, and infantile hyperostosis.

Nonhereditary nature and preponderance to affect the maxilla is a definite criterion to rule out fibrous dysplasia. Besides, the lesions are localized asymmetrically and present at a later stage between 15 and 30 yrs of age. They also lack the typical “cherubic” look. Central giant cell granuloma has indistinguishable histologic features from cherubism but they have a predilection to involve the anterior mandible and are rarely bilaterally symmetrical. The age predilection also is 10–30 yrs. Brown tumour of the jaws in hyperparathyroidism can be differentiated from cherubism by analysing blood chemistry. True giant cell tumour has a late age of presentation. Infantile hyperostosis in patients is characterised by acute inflammation of tissues accompanied by systemic changes and shows reactive new bone formation rather than lytic lesions. Other lesions like osteosarcoma [3], Langerhans cell histiocytosis, multiple odontogenic keratocysts, and fibrous osteoma show definitive variations from the classic features of cherubism.

The typical clinical, radiologic, and histologic features with familial incidence have led us to the conclusion of cherubism in these cases. Literature indicates that the state of normalisation is reached faster in patients with first degree than higher grades of cherubism [5]. The lesion was conservatively approached [8] because of the classic nature of the disease and allows natural involution to take place. Routine follow-up for two years has shown a decrease in the clinical size of the lesion.

## 4. Conclusion

The purpose of the paper is to report a rare hereditary case of cherubism which was conservatively treated and to add a note on its differential diagnosis.
